# Cytokine-induced killer cell/dendritic cell combined with cytokine-induced killer cell immunotherapy for treating advanced gastrointestinal cancer

**DOI:** 10.1186/s12885-020-06860-y

**Published:** 2020-04-28

**Authors:** Hansong Du, Jia Yang, Ying Zhang

**Affiliations:** 1 Department of Gastrointestinal Surgery, The Central Hospital of Wuhan, Tongji Medical College, Huazhong University of Science and Technology, Wuhan, China; 2 Department of Biochemistry & Molecular Biology, School of Basic Medicine, Tongji Medical College, Huazhong University of Science and Technology, Wuhan, China

**Keywords:** Advanced gastrointestinal cancer, Cytokine-induced killer, Dendritic cell, Meta-analysis, Systematic review

## Abstract

**Background:**

This study aimed to investigate the efficacy and safety of cytokine-induced killer (CIK)/dendritic cell combined with CIK (DC–CIK) cell therapy in advanced gastrointestinal cancer (GIC).

**Methods:**

The PubMed, Cochrane library, and Embase were searched to conduct a meta-analysis of clinical controlled trials to evaluate the efficacy and safety of CIK/DC–CIK cell therapy in advanced GIC. The pooled risk ratios (RRs) or weighted mean difference (WMD) with 95% confidence intervals (95% CIs) were calculated.

**Results:**

A total of nine studies with 1113 patients were identified. The overall survival (RR = 1.84, 95% CI = 1.41–2.40, *P*_heterogeneity_ = 0.654, *I*^2^ = 0%), progression-free survival (RR = 1.99, 95% CI = 1.52–2.60, *P*_heterogeneity_ = 0.727, *I*^2^ = 0%), and quality of life (WMD = 16.09, 95% CI = 1.66–30.52, *P*_heterogeneity_ < 0.001, *I*^2^ = 98.8%) were significantly improved in patients who received chemotherapy combined with CIK/DC–CIK cells, and no severe adverse events were reported.

**Conclusion:**

This meta-analysis suggested that the combination of CIK/DC–CIK immunotherapy and chemotherapy was safe and applicable for patients with advanced GIC. It is a feasible choice to prolong survival and improve quality of life.

## Background

Gastrointestinal cancers (GICs) are one of the leading causes of cancer-related mortality in the world, which majorly include cancers of colorectum and stomach (CRC and GC). In addition to growth and aging of global population, behavioral risk factors, such as smoking and dietary patterns, played an important role in the rising global burden of GIC [1]. Many treatment strategies, such as surgery, neoadjuvant chemotherapy, and adjuvant chemotherapy [2], are available for GIC patients. Moreover, early GIC patients can be cured by appropriate treatment, and the 5-year overall survival (OS) rate is 90%; however, for patients with advanced-stage GIC, the 5-year OC rate is still very low [3, 4].

Surgery, radiotherapy, and chemotherapy are routine treatments for GIC in most patients; they have the efficacy of eliminating and destroying primary tumors. However, these traditional treatments are often ineffective for advanced-stage cancers [5]. Cancer immunotherapy is a promising treatment method, which has made great progress in improving anti-tumor immunity [6]. Autoimmune therapy for malignant tumors is considered to be a feasible method, which mainly depends on the interference and inhibition effects of killer cells induced by the tumor, infiltrating lymphocytes and lymphokines, and CD3 monoclonal antibody [7, 8]. Dendritic cells (DCs) have the ability to present antigens, making them an attractive vehicle for the delivery and development of therapeutic tumor vaccines [9] . Cytokine-induced killer cells (CIK) is a heterogeneous effector CD3 + CD56 + NKT cell population that can be expanded from peripheral blood mononuclear cells (PBMC) in vitro [10, 11]. CIK cells have a strong MHC non-restrictive cytotoxicity, which inhibits both blood and solid malignancies, identifying and killing tumor targets without exposure or initiation. At present, CIK therapy or DC–CIK cell co-cultivation has been broadly applied in clinical trials for the treatment of GICs [12–20]; however, the results are not entirely consistent. Therefore, we conducted a systematic review and meta-analysis of published literature to evaluate the efficacy and safety of CIK/DC–CIK cells combined with chemotherapy for the treatment with advanced GIC cancer.

## Methods

### Literature search

The databases PubMed, Cochrane library, and Embase were searched for all relevant studies published in English. The search terms included “dendritic cells,” “immunotherapy,” “cytokine induced killer cells” OR “DC–CIK” combined with “colon OR rectal OR colorectal OR gastric cancer/tumor/carcinoma/neoplasm.” The last research was updated on January 1, 2019. The reference lists of all retrieved studies and published reviews were searched manually for additional references, and all identified relevant studies were included.

### Study selection

The inclusion criteria were as follows: (1) clinical controlled trials of patients with CRC or GC; (2) patients pathologically diagnosed with Tumor Node Metastasis (TNM) stages of II, III, and IV; and (3) patients in the experimental group treated using chemotherapy combined with CIK or DC–CIK immunotherapy, whereas patients in the control group treated using chemotherapy alone. The exclusion criteria were as follows: (1) reviews, conference abstracts, letter, or case reports; (2) multiple studies published on the same population (in which case the most recent and complete study was included); and (3) studies without available data for statistics. The studies meeting at least one of the aforementioned three criteria were excluded.

### Data extraction and quality assessment

The following data from each study were extracted independently by two authors: authors, year of publication, sample size, chemotherapy regimen, follow-up period, curative effect, and adverse events of each eligible trial. The primary endpoints were OS and progression-free survival (PFS). The other endpoints were complete response (CR), partial response (PR), overall response rate (ORR), and quality of life (QOL). Safety analyses were also performed. Two reviewers independently extracted the studies. Any disagreements were resolved by consensus. Evaluation of the research quality was managed using the Cochrane Collaboration’s tool for assessing risk of bias.

### Statistical analysis

The meta-analysis was conducted using Stata 14.0 (Stata Corp., TX, USA). Risk ratios (RRs) or weighted mean difference (WMD) with 95% confidence intervals (CIs) were calculated as effect sizes. RR was the effect measurement for dichotomous outcomes, while WMD was applied for the continuous variables. The potential heterogeneity across studies was examined via Cochran’s Q-statistic and *I*^2^ statistics. The *P* value for heterogeneity < 0.1 indicated that the heterogeneity was statistically significant. Thus, the random-effects model was used to perform the analysis. Otherwise, the summary effect was computed using the fixed-effects model. In the sensitivity analysis, the influence of each study on the summary effect was analyzed by dropping one study at a time. The Begg’s and Egger’s tests were conducted to evaluate publication bias. The trim-and-fill method was used to determine the effect of potential publication bias on the pooled estimates. A *P* value less than 0.05 was considered to be statistically significant.

## Results

### Literature search and study selection

**Fig. 1:**
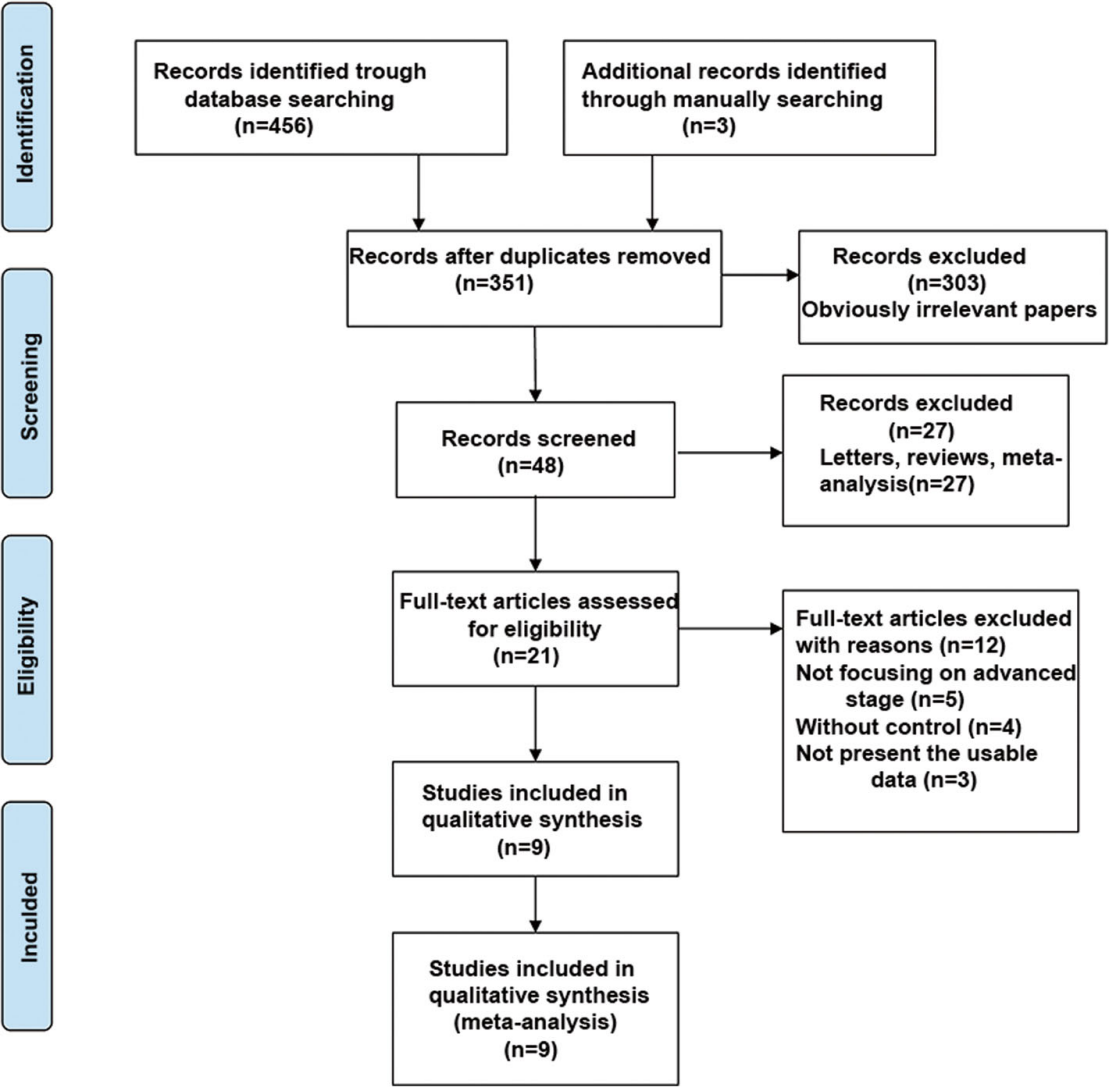
Flow diagram of study identification

A total of 466 studies were identified from PubMed, Cochrane library, and Embase. Then, three additional records were found by manually searching the reference lists of other studies. After deleting the duplications, 351 studies were selected. Then, 330 studies were discarded because of their irrelevance to the topic of interest. Of the remaining 21 studies, 3 were excluded for unavailability of data for statistics and four were excluded as they did not include controls. Besides, five studies did not focus on the advanced-stage GIC. Finally, a total of nine studies, including 1113 patients, met the inclusion and exclusion criteria in this meta-analysis^11–19^. The flow diagram of the search process is shown in Fig. 1.

### Study characteristics

**Fig. 2:**
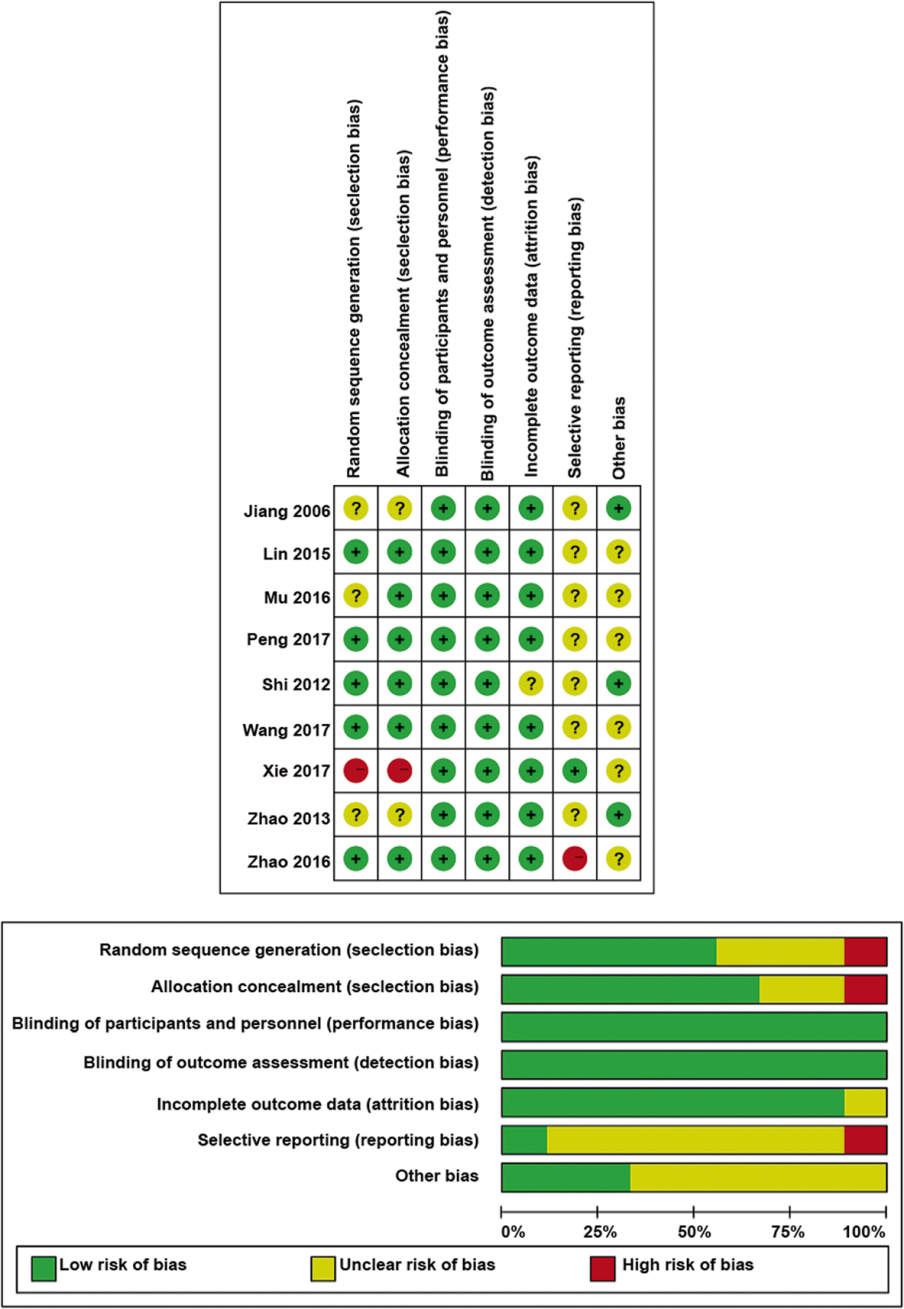
Risk-of-bias assessments for the randomized trials included in the meta-analysis. **a** Risk-of-bias summary. **b** Risk-of-bias graph. *Symbols*: (+), Low risk of bias; (?), unclear risk of bias; and (−), high risk of bias

**Fig. 3:**
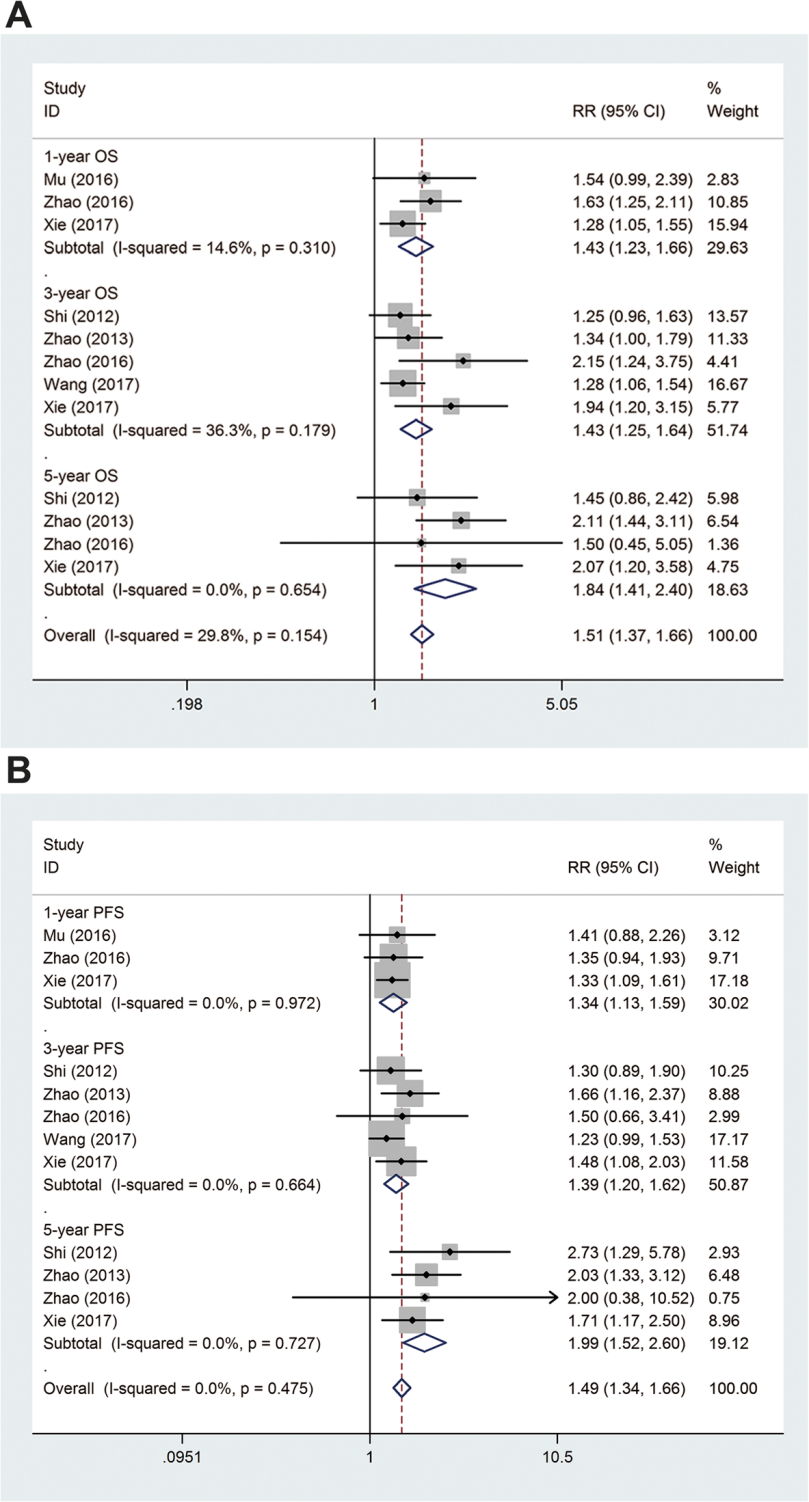
Forest plot of the comparison of survival. **a** Overall survival (OS). **b** Progression-free survival

The key characteristics of all included studies are summarized in Table 1. All the studies involved patients with advanced GIC followed up for at least 24 months. Nine studies from 2006 to 2017 compared CIK/DC–CIK plus chemotherapy with chemotherapy alone for treating advanced GIC. These nine studies were assessed using the Cochrane Collaboration’s tool for the risk of bias. A graph and summary of the risk of bias are shown in Figs. 2 and 3. Four studies did not mention randomization, and three studies did not provide information of allocation concealment.

**Table 1:** Characteristics of the studies included in this meta-analysis

Gender (Male/Female)	Mean age (Y)	Treatment design		Follow-up (M)	Study design	Stages	Outcomes assessed	
		Exp	Con					
Jiang/200 6[11]	Exp: 21/11; Con: 18/7	Exp: 54; Con: 52	CIK + FOLFOX4 (*N* = 32)	FOLFOX4 (*N* = 25)	60	Prospective	GC: IV	CR, PR, ORR, QOL
Shi/201 2[12]	Exp: 43/31; Con: 58/19	Exp: 58 ± 2.1; Con: 56 ± 1.5	CIK + COD (*N* = 74)	COD (*N* = 77)	80	Retrospective	GC: III, IV	OS, PFS
Zhao/201 3[13]	Exp: 40/13; Con: 87/25	NA	CIK + FUP or FOLFOX4 (*N* = 53)	FUP or FOLFOX4 (*N* = 112)	120	Retrospective	GC: II, III	OS, PFS
Lin/201 5[14]	Exp: 76/58; Con: 64/57	NA	DC–CIK + 5-FU, FOLFOX/XELOX (*N* = 134)	5-FU, FOLFOX/XELOX (*N* = 121)	50	Prospective	CRC: KPS score > 60	Thrombocytopenia, nausea, vomiting, abnormal liver function
Mu/201 6[15]	Exp: 10/3; Con: 10/5	NA	CIK + FOLFOX4/DCF (*N* = 13)	FOLFOX4/DCF (*N* = 15)	24	Prospective	GC: III, IV	OS, PFS, CR, PR, ORR, thrombocytopenia, nausea, vomiting, neutropenia
Zhao/201 6[16]	Exp: 41/20; Con: 41/20	Exp: 58; Con: 60	CIK + FOLFOX4 (*N* = 61)	FOLFOX4 (*N* = 61)	72	Prospective	CRC: KPS score > 70	OS, PFS, CR, PR, ORR, neutropenia
Peng/201 7[17]	Exp: 14/9; Con: 15/8	Exp: 52.2 ± 7.9; Con: 51.4 ± 8.5	CIK + FOLFOX4(*N* = 23)	FOLFOX4 (*N* = 23)	50	Prospective	CRC: II, III	QOL, nausea, vomiting, abnormal liver function, myelosuppression
Wang/201 7[18]	Exp: 38/13; Con: 73/23	NA	CIK + FP (*N* = 51)	FP (*N* = 96)	80	Prospective	GC: II, III	OS, PFS, myelosuppression
Xie/201 7[19]	Exp: 39/32; Con: 40/31	Exp: 55.3 ± 14.6; Con: 55.6 ± 14.3	DC-CIK + FOLFIRI (*N* = 71)	FOLFIRI (*N* = 71)	120	Retrospective	CRC: III, IV	OS, PFS, QOL

*CIK* Cytokine-induced killer biotherapy, *COD* cisplatin, oxaliplatin, and docetaxel, *Con* control group, *CR* complete response, *DC* dendritic cell, *DCF* docetaxel, cisplatin, 5-florouracil, *Exp* experimental group, *FOLFIRI* irinotecan (CPT-11), leucovorin (LV), and 5-FU regimen, *FOLFOX4* 5-fluorouridine, leucovorin, and oxaliplatin, *FP* 5-fluorouracil- and platinum, *KPS* Karnofsky performance status, *M* months, *NA* Not available, *ORR* overall response rate, *OS* overall survival, *PFS* progression-free survival, *PR* partial response, *QOL* quality of life, *Y* years

### Meta-analyses

#### Efficacy

Clinical responses were assessed in terms of the OS and PFS to evaluate the prognosis.

Among the nine trials, three reported 1-year OS rate, five reported 3-year OS rate, and four reported 5-year OS rate (Fig. 3a). As a slightly significant heterogeneity was detected, the fixed-effects model was used. Chemotherapy combined with CIK/DC–CIK immunotherapy showed a significant increase in 1-year OS (3 studies, 292 patients, RR = 1.43, 95% CI = 1.23–1.66, *P*_heterogeneity_ = 0.310, *I*^2^ = 14.6%), 3-year OS (5 studies, 727 patients, RR = 1.43, 95% CI = 1.25–1.64, *P*_heterogeneity_ = 0.179, *I*^2^ = 36.3%), and 5-year OS (4 studies, 580 patients, RR = 1.84, 95% CI = 1.41–2.40, *P*_heterogeneity_ = 0.654, *I*^2^ = 0%) compared with those of chemotherapy alone.

In terms of PFS, three studies presented relevant data of 1-year PFS, five reported 3-year PFS, and four reported 5-year PFS. As shown in Fig. 3b, chemotherapy combined with immunotherapy significantly prolonged 1-year PFS (3 studies, 292 patients, RR = 1.34, 95% CI = 1.13–1.59, *P*_heterogeneity_ = 0.972, *I*^2^ = 0%), 3-year PFS (5 studies, 727 patients, RR = 1.39, 95% CI = 1.20–1.62, *P*_heterogeneity_ = 0.664, *I*^2^ = 0%), and 5-year PFS (4 studies, 580 patients, RR = 1.99, 95% CI = 1.52–2.60, *P*_heterogeneity_ = 0.727, *I*^2^ = 0%) compared with chemotherapy alone.

#### Efficacy

**Fig. 4:**
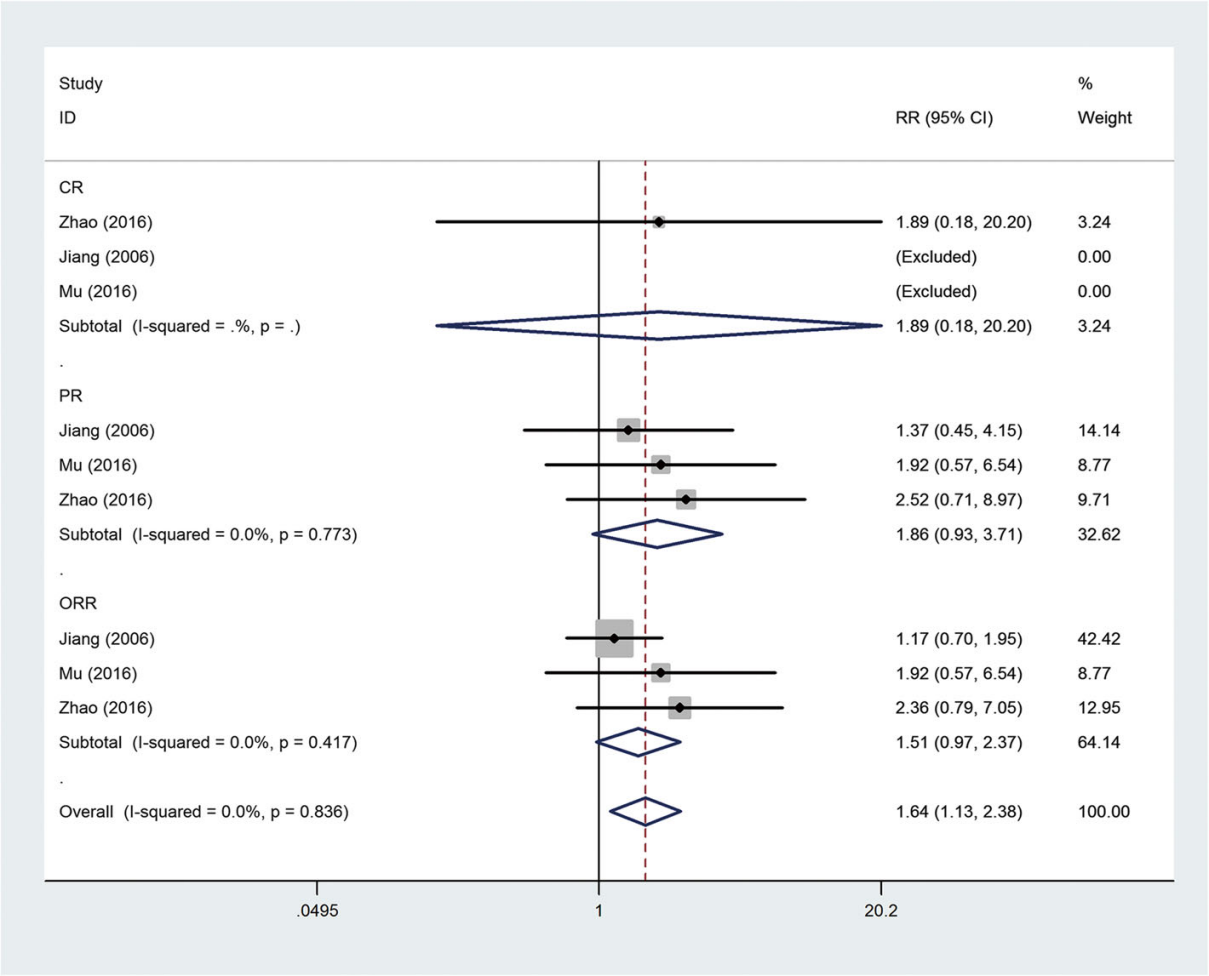
Forest plot of the comparison of complete response (CR), partial response (PR), and overall response rate (ORR)

Efficacy was assessed in terms of CR, PR, and ORR. The analysis result is shown in Fig. 4; however, the RR of CR (3 studies, 207 patients, RR = 1.89, 95% CI = 0.18–20.20), PR (3 studies, 207 patients, RR = 1.86, 95% CI = 0.93–3.71, *P*_heterogeneity_ = 0.773, *I*^2^ = 0%), and ORR (3 studies, 207 patients, RR = 1.51, 95% CI = 0.97–2.37, *P*_heterogeneity_ = 0.417, *I*^2^ = 0%) did not infer significant difference between the two groups. Three studies evaluated the effect of CIK/DC–CIK immunotherapy on the QOL of patients with advanced GIC. Significantly improved QOL was found in the CIK/DC–CIK immunotherapy group compared with the chemotherapy-alone group (3 studies, 245 patients, WMD = 16.09, 95% CI = 1.66–30.52, *P*_heterogeneity_ < 0.001, *I*^2^ = 98.8%) (Figure S1).

The safety of CIK/DC–CIK therapy in the treatment of advanced GIC was evaluated in this meta-analysis. As shown in Figure S2, no significant difference was observed in terms of thrombocytopenia (2 studies, 283 patients, RR = 1.04, 95% CI = 0.93–1.16, *P*_heterogeneity_ = 0.497, *I*^2^ = 0%), nausea, vomiting (3 studies, 329 patients, RR = 0.67, 95% CI = 0.35–1.28, *P*_heterogeneity_ = 0.003, *I*^2^ = 82.9%), abnormal liver function (2 studies, 301 patients, RR = 1.08, 95% CI = 0.95–1.23, *P*_heterogeneity_ = 0.373, *I*^2^ = 0%), neutropenia (2 studies, 150 cases, RR = 0.55, 95% CI = 0.31–1.00, *P*_heterogeneity_ = 0.955, *I*^2^ = 0%), and myelosuppression (2 studies, 193 patients, RR = 0.73, 95% CI = 0.48–1.11, *P*_heterogeneity_ = 0.088, *I*^2^ = 65.7%) between the two groups.

#### Subgroup analysis

This study probed the detailed results in subgroup analyses stratified by cancer type (GC or CRC). All subgroup results were quite consistent with the overall results. The results are summarized in Table 2.

**Table 2:** Subgroup analysis based on cancer type

Outcome	Subgroup	Number of trials	Effect (95%CI)	Overall effect estimate	Heterogeneity
1-Year OS	GC	1	1.54 (0.99, 2.39)	*P* = 0.056	*–*
	CRC	2	1.42 (1.21, 1.66)	*P* < 0.001	*I*^2^ = 54.1%, *P* = 0.140
3-Year OS	GC	3	1.29 (1.12, 1.48)	*P* < 0.001	*I*^2^ = 0%, *P* = 0.937
	CRC	2	2.03 (1.41, 2.93)	*P* < 0.001	*I*^2^ = 0%, *P* = 0.782
5-Year OS	GC	2	1.79 (1.31, 2.45)	*P* < 0.001	*I*^2^ = 26.8%, *P* = 0.242
	CRC	2	1.94 (1.18, 3.20)	*P* = 0.009	*I*^2^ = 0%, *P* = 0.634
1-Year PFS	GC	1	1.41 (0.88, 2.26)	*P* = 0.155	*–*
	CRC	2	1.33 (1.11, 1.60)	*P* = 0.002	*I*^2^ = 0%, *P* = 0.940
3-Year PFS	GC	3	1.36 (1.14, 1.61)	*P* = 0.001	*I*^2^ = 0%, *P* = 0.377
	CRC	2	1.49 (1.10, 2.01)	*P* = 0.010	*I*^2^ = 0%, *P* = 0.980
5-Year PFS	GC	2	2.25 (1.54, 3.29)	*P* < 0.001	*I*^2^ = 0%, *P* = 0.493
	CRC	2	1.73 (1.19, 2.52)	*P* = 0.004	*I*^2^ = 0%, *P* = 0.854
PR	GC	2	1.58 (0.70, 3.59)	*P* = 0.275	*I*^2^ = 0%, *P* = 0.685
	CRC	1	2.52 (0.71, 8.97)	*P* = 0.154	*–*
ORR	GC	2	1.30 (0.81, 2.09)	*P* = 0.278	*I*^2^ = 0%, *P* = 0.457
	CRC	1	2.36 (0.79, 7.05)	*P* = 0.124	*–*
QOL	GC	1	28 (26.36, 29.64)	*P* < 0.001	*–*
	CRC	2	9.32 (6.96, 11.69)	*P* < 0.001	*I*^2^ = 0%, *P* = 0.373

*CRC* Colorectal cancer, *GC* gastric cancer, *ORR* overall response rate, *OS* overall survival, *PFS* progression-free survival, *PR* partial response, *QOL* quality of life

#### Sensitivity analysis

**Fig. 5:**
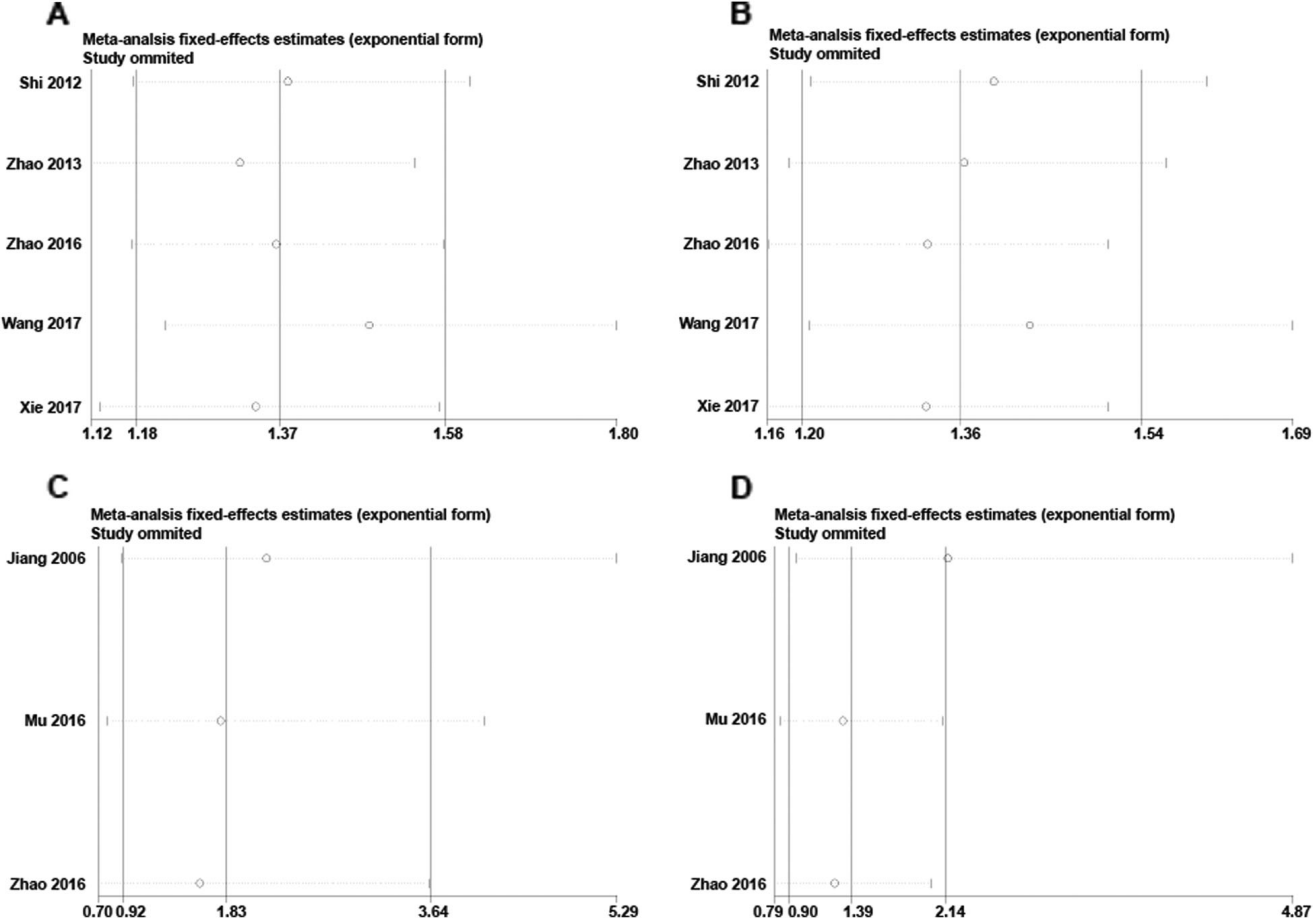
Sensitivity analysis examining the influence of individual study on pooled results. **a** Overall survival. **b** Progression-free survival. **c** Partial response. **d** Overall response rate

Sensitivity analyses were performed to assess the stability of the results by sequentially removing each study. The removal of any single study did not change the overall statistical results, indicating that the results of this study were statistically robust (Fig. 5).

#### Publication bias

**Fig. 6:**
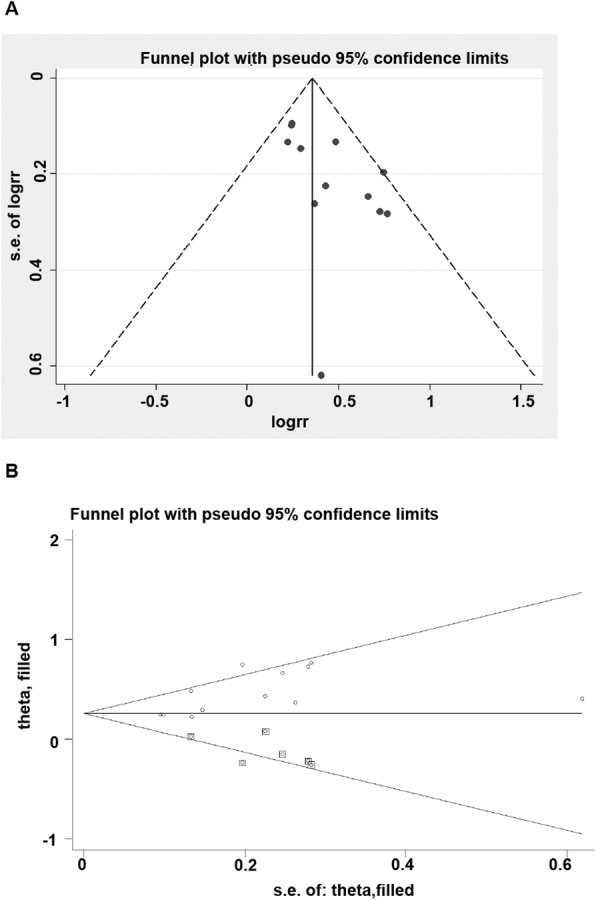
Funnel plot for publication bias. Each point represents a separate study for the indicated association. **a** Funnel plot of OS. **b** Trim-and-fill plot of OS

The outcome of OS, with the largest number of included studies, was chosen to test the publication bias. Visual examination of the funnel plot (Fig. 6a) revealed a considerable degree of asymmetry. In addition, publication bias was statistically significant by Egger’s test or Begg’s test (Begg’s test, *P* = 0.034; Egger’s test, *P* = 0.016). Therefore, a sensitivity analysis was conducted using the trim-and-fill method (Fig. 6b) [21]. After imputing six unpublished studies, the trim and fill sensitivity analysis did not change the general result (RR = 0.255, 95% CI = 0.176–0.333, *P* < 0.01).

## Discussion

GIC is still a fatal threat to human health due to metastasis, with recurrence inducing refractory advanced tumor stage and poor prognosis. Yan et al. [22] proved that the recurrence rate of patients with GC ranged from 40 to 65% due to distant metastasis and local recurrence. Adoptive cellular immunotherapy can be used in combination with standard therapy as adjuvant and palliative treatment after operation to improve the survival rate and QOL of patients with GIC. The CIK cells have shown the best efficacy in this treatment. Compared with lymphokine-activated killer cells, CIK cells are more readily available and show higher tumor-specific cytotoxic activity [23–27]. To date, several clinical trials have used chemotherapy plus DC–CIK immunotherapy to treat advanced GIC. However, in these trials, the clinical protocol applied was not standard, blurring the evaluation of treatment effects. In this study, a large number of comprehensive trials were investigated to achieve higher statistical reliability. This meta-analysis showed that chemotherapy combined with CIK/DC–CIK immunotherapy improved the OS, PFS, and QOL without causing serious adverse events.

This study confirmed the safety of CIK/DC–CIK immunotherapy for advanced GIC patients, and the adverse events caused were tolerated by all patients. No significant difference was observed in terms of common adverse events, such as thrombocytopenia (RR = 1.04, 95% CI = 0.93–1.16, *P*_heterogeneity_ = 0.497, *I*^2^ = 0%), nausea, vomiting (RR = 0.67, 95% CI = 0.35–1.28, *P*_heterogeneity_ = 0.003, *I*^2^ = 82.9%), abnormal liver function (RR = 1.08, 95% CI = 0.95–1.23, *P*_heterogeneity_ = 0.373, *I*^2^ = 0%), neutropenia (RR = 0.55, 95% CI = 0.31–1.00, *P*_heterogeneity_ = 0.955, *I*^2^ = 0%), and myelosuppression (RR = 0.73, 95% CI = 0.48–1.11, *P*_heterogeneity_ = 0.088, *I*^2^ = 65.7%) in the CIK/DC–CIK immunotherapy group compared with the chemotherapy-alone group. The CIK/DC–CIK immunotherapy enhanced the efficiency of conventional therapy in treating advanced GIC. Compared with the conventional therapy group, the 1-year OS, 3-year OS, 5-year OS, 1-year PFS, 3-year PFS, and 5-year PFS of patients in the combination therapy group improved remarkably. Moreover, the combination therapy improved the QOL (WMD = 16.09, 95% CI = 1.66–30.52, *P*_heterogeneity_ < 0.001, *I*^2^ = 98.8%) of the patients by relieving pain, decreasing fatigue and insomnia, and improving appetite.

Chemotherapy is thought to damage the immune system. Immunotherapy is a type of cancer treatment that helps the body’s own immune system fight cancer, many clinical trials of immunotherapy have been conducted in multiple centers. DCs are an important immunotherapeutic cell type. Ishigami et al. [28] demonstrated that patients with GC having a high level of DC cell infiltration were less likely to have lymph node metastasis and a significantly increased 5-year survival rate. In addition, CIK cells exhibit strong cytotoxicity against a variety of tumor cell lines as well as newly isolated tumor samples (e.g. liver cancer [29], lung cancer [30], glioma [31], and GC [32]). Compared with other immune cells, CIK cells proliferate rapidly and exhibit strong antitumor activity and a broad spectrum of targeted tumors [14, 33]. Therefore, CIK/DC–CIK cell-based immunotherapy is a promising treatment for a number of cancers. Zhao et al. [34] showed that “GP regimen combined with DC–CIK immunotherapy could reduce postoperative recurrence and prolong survival in patients with non-small-cell lung cancer.” Ma et al. [35] demonstrated that CIK cell therapy had significant advantages in prolonging the median OS rate, PFS, DCR, ORR, and QOL of patients with hepatocellular carcinoma. Other studies of gastric cancer [13], advanced renal cancer [36], and metastatic nasopharyngeal carcinoma [37] demonstrated that DC–CIK cells could improve the prognosis of patients. CIK cells have become a promising immunotherapy method in tumor therapy due to their easy availability and strong antitumor activity, which is of great significance for tumor prognosis [38].

Heterogeneity is a problem with most meta-analyses. In the present meta-analysis, heterogeneity was found in overall analyses; thus, the random-effects model was used. Sensitivity analysis was performed to assess the stability of the results by sequentially removing each study. The removal of any single study did not change the overall statistical results, indicating that the results of this study were statistically robust. Furthermore, this study probed the detailed results in subgroup analyses stratified by cancer type (GC or CRC). Based on the data collected, this study suggested that the cancer type at least partly contributed to the between-study heterogeneity.

This meta-analysis had some limitations that might have affected the interpretation of results. First, the efficacy of CIK/DC–CIK immunotherapy was affected by many factors, such as injection mode, tumor stage, and metastasis cycle. Further, a detailed analysis needs to be carried out on the basis of complete literature, standardization of treatment options, and limitation of patient participation criteria. Second, some data on adverse events could only be used in two trials, which might have led to bias. Third, publishing bias existed in this study. Although the trim-and-fill method was used in this study to confirm the results, some negative data that were possibly omitted might have affected the results. Finally, the heterogeneity could not be completely eliminated in the analysis.

## Conclusion

In summary, this meta-analysis confirmed that the combination of CIK/DC–CIK immunotherapy and chemotherapy was safe and applicable for patients with advanced GIC, and hence might serve a feasible choice to prolong survival and improve QOL. Therefore, CIK/DC–CIK immunotherapy is an effective therapy for advanced GIC treatment.

## Supplementary information

**Additional file 1: Figure S1.** Forest plot of the comparison of quality of life (QOL).

**Additional file 2: Figure S2.** Forest plot of the comparison of adverse events (AEs).

## Data Availability

The datasets used and/or analyzed during the current study are available from the corresponding author on reasonable request.

## Abbreviation

CIK: Cytokine-induced killer
DC–CIK: Dendritic cell combined with CIK
GIC: Cell therapy in advanced gastrointestinal cancer
WMD: Weighted mean difference
RRs: Risk ratios
CIs: Confidence intervals
OS: Overall survival
TNM: Tumor Node Metastasis
PFS: Progression-free survival
CR: Complete response
PR: Partial response
ORR: Overall response rate
QOL: Quality of life
