# Supplementary figures and images for: Cytokine-induced killer cell/dendritic cell combined with cytokine-induced killer cell immunotherapy for treating advanced gastrointestinal cancer
Source: BMC Cancer. 2020 Apr 28;20:357. doi: 10.1186/s12885-020-06860-y (PMC7189715; doi:10.1186/s12885-020-06860-y)

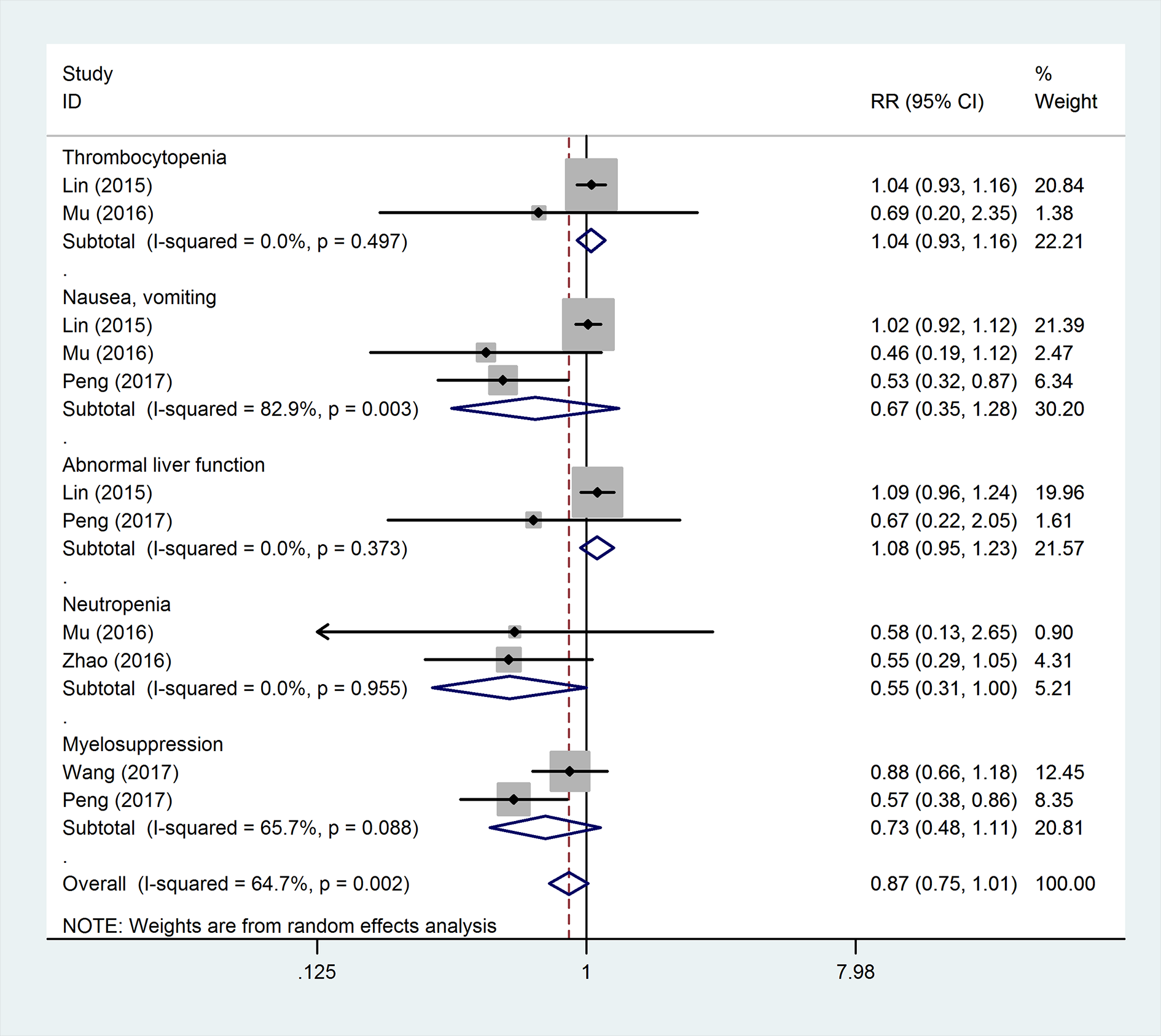

Supplement: Supplementary file 2 — Additional file 2: Figure S2. Forest plot of the comparison of adverse events (AEs). [file 12885_2020_6860_MOESM2_ESM.tiff]
